# Interval Timing in Pediatric Multiple Sclerosis: Impaired in the Subsecond Range but Unimpaired in the One-Second Range

**DOI:** 10.3389/fneur.2020.575780

**Published:** 2020-10-20

**Authors:** Stefan J. Troche, Tugba Kapanci, Thomas H. Rammsayer, Carl P. A. Kesseler, Martin Georg Häusler, Tobias Geis, Mareike Schimmel, Christiane Elpers, Jonas H. Kreth, Charlotte Thiels, Kevin Rostásy

**Affiliations:** ^1^Institute of Psychology, University of Bern, Bern, Switzerland; ^2^Department of Psychology and Psychotherapy, University of Witten/Herdecke, Witten, Germany; ^3^Division of Neuropediatrics and Social Pediatrics, Department of Pediatrics, University Hospital, Rheinisch-Westfälische Technische Hochschule Aachen, Aachen, Germany; ^4^Department of Pediatric Neurology, Klinik St. Hedwig, University Children's Hospital Regensburg (Kinder-Universitätsklinik Ostbayern KUNO), Regensburg, Germany; ^5^Pediatric Neurology, Children's Hospital, University Hospital Augsburg, Augsburg, Germany; ^6^Neuropediatric Department, Children's University Hospital Muenster, Muenster, Germany; ^7^Department of Pediatric Neurology, Hospital for Children and Adolescents, Klinikum Leverkusen, Leverkusen, Germany; ^8^Department of Pediatrics and Pediatric Neurology, Ruhr University Bochum, Bochum, Germany; ^9^Pediatric Neurology, University of Witten/Herdecke, Children's Hospital Datteln, Datteln, Germany

**Keywords:** cognitive impairment, interval timing, pediatric multiple sclerosis (MS), neuropsychology, distinct timing hypothesis, temporal information processing

## Abstract

**Background:** For adult multiple sclerosis (MS) patients, impaired temporal processing of simultaneity/successiveness has been frequently reported although interval timing has been investigated in neither adult nor pediatric MS patients. We aim to extend previous research in two ways. First, we focus on interval timing (instead of simultaneity/successiveness) and differentiate between sensory-automatic processing of intervals in the subsecond range and cognitive processing of intervals in the one-second range. Second, we investigate whether impaired temporal information processing would also be observable in *pediatric* MS patients' interval timing in the subsecond and one-second ranges.

**Methods:** Participants were 22 pediatric MS patients and 22 healthy controls, matched for age, gender, and psychometric intelligence as measured by the Culture Fair Test 20-R. They completed two auditory interval-timing tasks with stimuli in the subsecond and one-second ranges, respectively, as well as a frequency discrimination task.

**Results:** Pediatric MS patients showed impaired interval timing in the subsecond range compared to healthy controls with a mean difference of the difference limen (DL) of 6.3 ms, 95% CI [1.7, 10.9 ms] and an effect size of Cohen's *d* = 0.830. The two groups did not differ significantly in interval timing in the one-second range (mean difference of the DL = 26.9 ms, 95% CI [−14.2, 67.9 ms], Cohen's *d* = 0.399) or in frequency discrimination (mean difference of the DL = 0.4 Hz, 95% CI [−1.1, 1.9 Hz], Cohen's *d* = 0.158).

**Conclusion:** The results indicate that, in particular, the sensory-automatic processing of intervals in the subsecond range but not the cognitive processing of longer intervals is impaired in pediatric MS patients. This differential pattern of results is unlikely to be explained by general deficits of auditory information processing. A tentative explanation, to be tested in future studies, points to subcortical deficits in pediatric MS patients, which might also underlie deficits in speech and visuomotor coordination typically reported in pediatric MS patients.

## Introduction

Multiple sclerosis (MS) is an inflammatory neurological disease, which leads to demyelination and neuroaxonal injury of the central nervous system and, subsequently, to physical and cognitive impairments. In about 5% of MS patients, onset of the disease is before the age of 18 years (1), and age of onset plays a crucial role in individual differences in neurological and cognitive effects of MS (2). According to Charvet et al. (3), one third of pediatric MS patients suffer from cognitive impairment already in the early phase of the disease. In line with this observation, children and adolescents with MS suffer from substantial brain volume loss already at the time of the first event (4). Pediatric and adult MS patients seem to differ in their cognitive deficits (5), and a longitudinal cohort study demonstrates a more pronounced decline of information-processing efficiency for individuals with pediatric- than adult-onset MS, primarily at the age of about 30 (6). Probably due to the low prevalence of pediatric MS, the manifoldness of cognitive impairments is less well-investigated in pediatric compared to adult MS patients. For example, processing of temporal information has been reported to be impaired in adult MS patients but, to the best of our knowledge, has not been investigated in pediatric MS patients yet.

Temporal information processing does not represent a unitary concept but rather consists of distinct elementary temporal experiences [e.g., (7, 8)]. Researchers interested in the functional relationship between temporal information processing and brain functioning have devoted particular attention to two elementary time experiences: (1) simultaneity and successiveness and (2) interval timing.

Investigations into *simultaneity* and *successiveness* are concerned with the size of the temporal interval between two or more events that is required for them to be perceived as separate events (successiveness) rather than fused as one event (simultaneity). Visual or auditory fusion thresholds, for example, represent an indicator of this type of temporal resolution power for central sensory information processing (9). Over the past six decades, a large number of clinical studies provide convincing evidence for MS patients' significantly impaired visual-temporal resolution ability as indicated by higher fusion thresholds compared to healthy controls (10–14). Although no auditory fusion studies in MS patients seem to exist, important clues for impaired auditory temporal resolution ability comes from a recent study by Valadbeigi et al. (15). As a tool for evaluating temporal resolution ability in MS patients, these authors assess gap detection thresholds. For this purpose, participants had to detect silent intervals ranging from 2 to 20 ms embedded in 6-s segments of white noise. MS patients showed significantly higher thresholds for gap detection than healthy controls, indicating impaired auditory temporal resolution performance in MS patients.

*Interval timing*, including time estimation and duration discrimination, refers to the accurate timing of events. Accurate timing plays a crucial role for motor processes (16), speech (17), and learning (18) as well as working memory functioning (19). Hence, interval timing can be considered a basic component of cognitive functioning of all sorts [cf. (20)]. Given the important role of timing processes for cognitive functions shown to be impaired in MS patients, it is very surprising that no studies on interval timing in pediatric MS patients seem to exist. The aim of the present study, therefore, was to investigate, for the first time, performance on interval timing tasks in MS patients by comparing a group of pediatric MS patients with a group of healthy controls matched for age, sex, and psychometric intelligence.

The so-called distinct timing hypothesis [cf. (21, 22)] suggests two dissociable mechanisms for the timing of extremely brief durations in the subsecond range and longer durations, respectively. More precisely, interval durations less than approximately 300–500 ms can be perceived directly due to sensory-automatic temporal processing, whereas the duration of longer intervals needs to be reconstructed by higher mental processes [cf. (22)]. To tap into performance differences between MS patients and healthy controls in both the sensory-automatic as well as the cognitive processes involved in interval timing, two auditory duration-discrimination tasks with base durations of 100 and 1,000 ms, respectively, were applied in the present study. Furthermore, in order to control for more general, non-temporal, MS-related deficits in sensory transmission of acoustic stimuli [e.g., (23)], we also employed a frequency-discrimination task in addition to the two timing tasks.

## Methods

### Participants

Twenty-three pediatric MS patients (19 females) participated in the present study. Their age ranged from 12 to 18 years (*M* ± *SD*: 15.6 ± 1.9 years). Mean age at disease onset was 14.3 (± 1.8) years, and the mean number of relapses was 2.61 (± 1.03). Scores on the Expanded Disability Status Scale (EDSS; (24)) ranged from 0 to 6.5 with a mean score of 1.65 (± 1.70), and their mean IQ was 97.43 (± 9.37) according to Cattell's Culture Fair Test 20-R (CFT 20-R). Diagnoses were based on the recently revised McDonald criteria (25). Twenty pediatric MS patients were treated with Interferon, two with Glatirameracetat, and one was therapy-naive. No patient received steroid treatment. Furthermore, no patient had clinical disease activity at the time of testing, and the attending doctors judged the clinical status of all participating patients as stable.

Previous research reveals that interval timing improves with increasing age of children and adolescents (26, 27) and that males might have lower discrimination thresholds than females (28). Furthermore, psychometric intelligence is positively related to performance on interval timing tasks (29) and has a differential effect on cognitive impairments due to MS (30). Therefore, the 23 MS patients were compared to 23 participants, out of a pool of 63 (neurologically and psychologically) healthy adolescents, matched for age, sex, and intelligence by means of a nearest-neighbor matching algorithm (31). The algorithm determined 19 female and four male healthy controls with a mean age of 16.4 (± 2.2) years and a mean IQ of 99.4 (± 10.7). They did not differ significantly from MS patients in age, *t*(43.007) = 1.373, *p* = 0.177, *d* = 0.405, and intelligence test scores, *t*(43.210) = 0.658, *p* = 0.514, *d* = 0.194.

All MS patients and healthy controls reported normal hearing and normal or corrected-to-normal vision. All participants and the parents of participants younger than 18 years were informed about the study protocol and signed informed consent prior to the study. The study was approved by the local ethics committee of the University of Witten/Herdecke (No. 173/2016).

### Assessment of Depression

With the German Depression Inventory for Children and Adolescents [DIKJ; (32)], the severity of major depression symptoms was measured. For each of the 29 items, children chose the most applicable statement out of three alternatives. Stiensmeier-Pelster et al. (32) report high reliability coefficients ranging between Cronbach's α = 0.87 and 0.92.

### Assessment of Fatigue

With the 21 items of the German Modified Fatigue Impact Scale [MFIS; (33)], MS patients and healthy controls self-reported the severity with which fatigue affected physical, cognitive, and psychosocial aspects of their lives. According to Fisk et al. (33), the internal consistency is α = 0.81. One MS patient did not respond to one and another patient did not respond to two MFIS items. Their sum scores were estimated on the basis of the other 20 or 19 items, respectively.

### Expanded Disability Status Scale (EDSS)

The severity of MS-related disability in patients at the time of data collection was assessed by means of the EDSS (24). Scores could range from 0 to 10.

### Experimental Tasks

The three experimental tasks employed in the present study have previously been validated for investigating interval timing and frequency discrimination in children and adolescents (26, 27). A Lenovo notebook (L540) was used with a 15" monitor as well as an external audio interface (Steinberg, UR22 MKII) and headphones (Sennheisser HDA300). Stimuli were presented by E-prime 2.0 experimental software and responses were given on a Cedrus® keyboard (RB-840).

### Interval Timing in the Subsecond Range

Stimuli were white noise bursts presented at an intensity of 68 dB. The task consisted of 64 trials. Each trial consisted of a constant 100-ms standard interval and a variable comparison interval presented with an interstimulus interval (ISI) of 900 ms. The order of standard and comparison intervals within a trial was balanced and randomized across trials. The participant's task was to decide whether the first or the second stimulus was of longer duration by pressing one of two designated keys. Visual feedback was given after the response on the monitor for 1,500 ms (a “+” after a correct and a “–” after an incorrect response). After an intertrial interval of 600 ms, the next trial started.

The 64 trials were assigned to two interleaved series. In one series, the comparison (with an initial duration of 65 ms) was shorter than the standard interval. In the other series, the comparison (with an initial duration of 135 ms) was longer than the standard interval. Using the adaptive weighted up–down method (34), the difference between the comparison and standard intervals decreased after a correct response (5 ms in the first six trials, 3 ms in the following trials) and increased after an incorrect response (15 ms in the first six trials, 9 ms in the following trials). With this step-size ratio of 1:3, the two series converged to the 25% difference threshold (series with comparison interval shorter than standard) and the 75% difference threshold (series with comparison interval longer than standard), which were estimated from the last 20 trials of each series. The difference limen [DL; (35)] was computed as individual performance score, which refers to half the difference of the 75% and 25% difference thresholds. With this measure, superior performance on duration discrimination is indicated by smaller DL values.

### Interval Timing in the One-Second Range

Hardware and software as well as the number of trials and the experimental procedure were the same as in the duration discrimination task in the range of milliseconds. The only differences were that the standard interval had a duration of 1,000 ms and the initial comparison intervals of 500 and 1,500 ms in the two series for the estimation of the 25% and the 75% difference thresholds. Step-sizes of the change of the comparison interval were 25 ms after a correct (100 ms in the first six trials) and 75 ms after an incorrect response (300 ms in the first six trials). Again, the DL was computed as individual performance score.

### Frequency Discrimination

The experimental procedure was the same as for the duration-discrimination tasks with the following exceptions. All stimuli were sine wave tones of 500 ms duration and presented with an intensity of 68 db. Each trial consisted of a standard tone with a frequency of 440 Hz and a comparison tone with a variable frequency and initial values of 438 Hz in the series converging to the 25% difference threshold and 442 Hz in the series converging to the 75% difference threshold. The step sizes were 0.3 Hz (0.5 Hz in the first six trials) after a correct response and 0.9 Hz (1.5 Hz in the first six trials) after an incorrect response. The ISI was 500 ms. The DL was computed as individual performance score.

### Assessment of Intelligence

The CFT 20-R (36), composed of three subtests (series, classifications, matrices) with 27 items, respectively, and one subtest (topologies) with 20 items, was administered individually and lasted about 1 h. The reliability of the CFT 20-R is high with *r*_tt_ = 0.96. Originally, the CFT was developed to assess fluid intelligence as an abstract reasoning ability independent from crystallized intelligence, which refers to language- and knowledge-related abilities. Thus, rather specific language deficits in MS patients do not (or only marginally) bias the assessment of intelligence by means of the CFT. The high correlation between CFT scores and general intelligence underlines its adequacy to measure an individual's overall cognitive functioning (37). The version CFT 20-R (36) is validated for adults and children and comprises fine-grained age-stratified IQ norms for children older than 6 years, adolescents, and adults. As a dependent variable, correct responses across all subtests were added to raw scores and transformed to age-stratified IQ equivalents.

### Time Course of the Study

The session started with verbal and written information about the study and signing informed consent by the participants and/or their parents followed by the administration of DIKJ and MFIS. The experimental part of the study started with two tasks, which lasted about 25 min and are reported in detail by Kapanci et al. (38). After a break of 15 min, the three discrimination tasks were presented in counterbalanced order. Each task lasted about 10 min. After another short break, participants completed the CFT 20-R. The total session lasted about 120 min.

## Results

An initial outlier detection revealed that discrimination thresholds in the interval timing task in the second range of one female MS patient and one female healthy control were more than three standard deviations above the mean of the respective group. These two participants were excluded from further analyses. Descriptive data as well as appropriate *t*-tests for age, IQ, depression, and fatigue are provided in Table 1 for the remaining 22 MS patients and 22 healthy controls of the final sample. MS patients and healthy controls did not differ significantly in age and IQ. Furthermore, no significant differences were obtained regarding symptoms of depression and fatigue.

**Table 1:** Mean (M) and standard deviation (SD) of age, normed CFT 20-R IQ scores, fatigue (MFIS), and depression scores (DIKJ) as well as difference limen (DL) in the two interval timing tasks and the frequency discrimination task for 22 pediatric MS patients and 22 healthy controls.

	**Pediatric MS**		**Healthy controls**		***t***	***df***	***P***	***d***
	***M***	***SD***	***M***	***SD***				
Age [years]	15.5	1.9	16.5	2.1	−1.644	41.635	0.108	−0.496
IQ	97.1	9.4	99.5	11.0	−0.781	41.083	0.440	−0.235
MFIS	30.7	18.1	23.5	10.5	1.632	33.623	0.112	0.492
DIKJ	13.2	6.0	12.8	6.3	0.246	41.875	0.807	0.074
DL of interval timing in the millisecond range [ms]	19.8	8.3	13.6	6.8	2.753	40.308	0.009	0.830
DL of interval timing in the second range [ms]	166.6	67.4	139.7	67.4	1.322	42.000	0.193	0.399
DL of frequency discrimination [Hz]	6.4	2.0	6.0	2.9	0.525	37.756	0.603	0.158

*Also reported are t-tests and corresponding effect sizes (Cohen's d)*.

**Figure 1:**
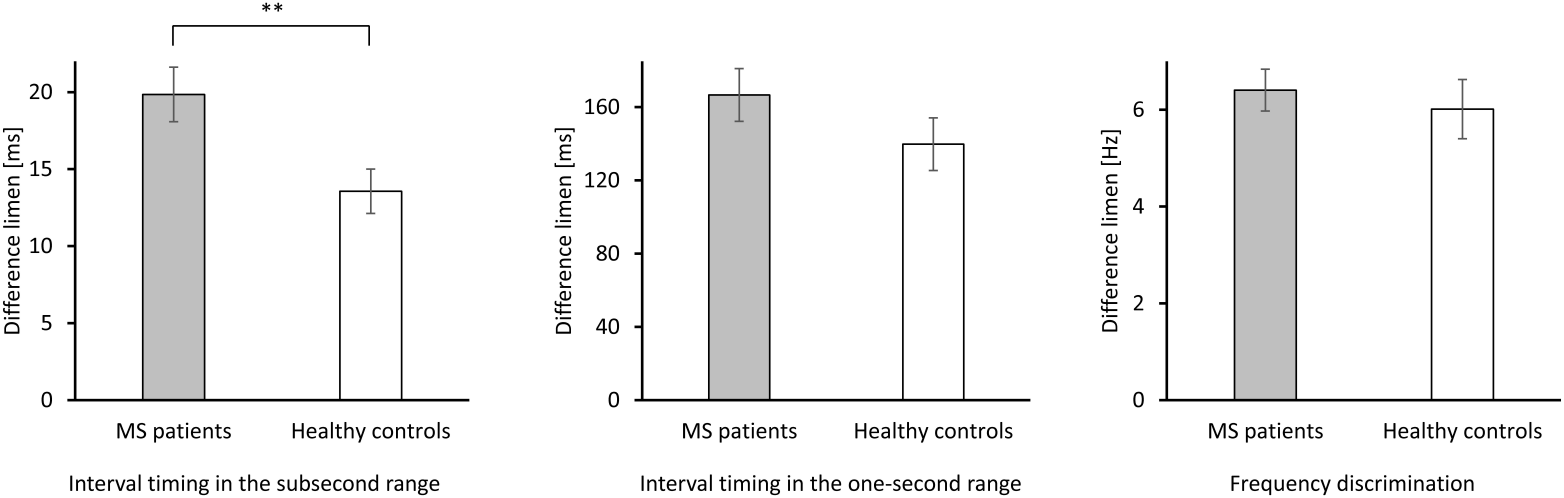
Performance as indicated by DL of 22 pediatric MS patients and 22 healthy controls in the two duration discrimination tasks (left panel: interval timing in the subsecond range; intermediate panel: interval timing in the one-second range) and in the frequency discrimination task (right panel). Smaller DL indicate better performance on the respective task. **p < 0.01 (two-tailed).

The main outcome variables of the present study were DL values in the two interval timing tasks (with stimuli in the subsecond and in the second range) and in the frequency discrimination task. Differences in discrimination performance, as indicated by DL values, between pediatric MS patients and healthy controls were investigated by means of three *t-*tests. In order to avoid alpha inflation, alpha was Bonferroni adjusted to α = 0.017. Descriptive statistics, results of *t*-tests, and effect sizes (Cohen's *d*) are reported in Table 1. As can be seen from Figure 1, MS patients differed significantly from healthy controls in their performance on neither the frequency discrimination task (mean difference in DL = 0.4 Hz; 95% [−1.1, 1.9 Hz]) nor the interval timing task with stimulus durations in the one-second range (mean difference in DL = 26.9 ms; 95% CI [−14.2, 67.9 ms]). For interval timing in the subsecond range, however, mean DL was significantly larger in pediatric MS patients than in healthy controls. The mean difference in DL was 6.3 ms with the 95% confidence interval not including zero [1.7, 10.9 ms]. This result indicated worse performance in pediatric MS patients compared to healthy controls as they needed larger differences between two durations in the subsecond range to correctly identify the longer one.

It should be noted that the same pattern of results was obtained when only the data of the 18 female participants in each group were analyzed. Furthermore, neither in MS patients nor in healthy controls was age significantly correlated with performance on the interval timing or the frequency discrimination tasks. Given that the two groups did not differ in age, a systematic influence of age on the above reported results is unlikely.

## Discussion

The aim of the present study was to investigate possible impairments of interval timing in pediatric MS patients using two auditory duration discrimination tasks that focused on interval timing in the subsecond and one-second ranges, respectively. Compared to healthy controls, MS patients showed impaired interval timing in the subsecond range but no significant differences in the one-second range. These differences in the subsecond range are unlikely to be based on general deficits of auditory information processing as the auditory demands regarding the duration discrimination task in the one-second range were virtually identical. Moreover, there were no differences in the frequency discrimination thresholds between MS patients and healthy controls. Due to the matching procedure, differences in age, sex, and psychometric intelligence can also be excluded to explain MS patients' impaired interval timing in the subsecond range.

Our findings expand previous results on impaired perception of simultaneity and successiveness in adult MS patients in two ways. First, timing deficits do not only occur in adult but also in pediatric MS patients. Second, in addition to judgments of simultaneity and successiveness as previously reported (13–15) MS also affects interval timing in the subsecond range—at least in pediatric patients.

Our findings support the distinct timing hypothesis (21, 22), which suggests two dissociable mechanisms underlying the timing of extremely brief durations in the subsecond range and longer durations in the second range. It appears that the sensory-automatic temporal processing of extremely brief durations below 300–500 ms is substantially impaired in pediatric MS patients, whereas cognitively mediated temporal processing of longer durations is less affected.

As depicted in Figure 1, MS patients show a performance decrement in both duration discrimination tasks compared to healthy controls. We cannot rule out that, in a larger sample, the difference in interval timing in the one-second range between MS patients and healthy controls might also have become statistically significant. The effect size, however, was more than twice as large for the interval timing in the subsecond compared to the one-second range. Thus, the sensory-automatic processes underlying the timing of intervals in the subsecond range seem to be particularly vulnerable to degenerative changes in the brain associated with MS.

There is good empirical evidence for the notion that distinct but partly overlapping neural networks underlie interval timing in the sub- and suprasecond range. In the meta-analysis by Wiener et al. (39), activation in the inferior frontal cortex, supplementary motor areas, precental gyrus, parietal lobe, insular cortex, claustrum, and putamen was related to both sub- and suprasecond timing. Particularly pronounced activation during temporal processing in the suprasecond range was found for the (right) prefrontal brain areas [see also (40)]. For timing in the subsecond range, specific activation was primarily identified in subcortical areas, such as the cerebellum (39, 41), thalamus, and striatal parts of the basal ganglia (39, 42) as well as some neocortical areas (e.g., the right inferior parietal lobe). Most interestingly, MS-related deficits in subcortical areas have been reported even at an early stage of the disease (43) and more frequently in pediatric than adult patients (44). Hence, a tentative explanation of the present findings might be that pediatric MS patients' impaired timing performance in the subsecond range is indicative of deficits in subcortical brain areas.

Previous research shows that accurate timing in the subsecond range plays an important role for motor coordination and visuomotor integration (16) and for speech perception and production (17) as well as speed of information processing (29). Against this background, it is particularly interesting that pediatric MS patients process information more slowly than healthy controls (5), have more problems integrating visuomotor information (1, 3, 45–47), and have deficits in fine motor coordination (46). Moreover, pediatric MS patients more frequently show receptive and expressive language deficits (1, 45, 47, 48). Thus, it would be promising for future research to investigate to what degree pediatric MS patients' timing deficits—as observed in the present study—is functionally related to their commonly observed deficits in motor coordination, processing speed, and speech. Such results would contribute to a better understanding of the neurocognitive mechanisms underlying MS patients' health-related restrictions observed in everyday life.

In sum, pediatric MS patients in the present study show impaired performance on interval timing in the subsecond range compared to healthy controls. This impairment is unlikely to be explained by auditory deficits because no performance differences between the two groups could be established for interval timing in the one-second range and frequency discrimination. As most brain areas specifically affecting interval timing in the subsecond range are subcortical, a tentative, but plausible explanation might point to subcortical alterations in the present sample of pediatric MS patients. Timing in the subsecond range is important for many daily life activities, such as visuomotor coordination or speech commonly impaired in pediatric MS patients. If future studies establish functional relationships between MS-related deficits in interval timing in the subsecond range and these daily life activities, the investigation of interval timing in the subsecond range might be a promising approach to better understand the underlying causes of these deficits.

## Data Availability Statement

The raw data supporting the conclusions of this article will be made available by the authors, without undue reservation.

## Ethics Statement

The studies involving human participants were reviewed and approved by local ethic committee of the University of Witten/Herdecke (No. 173/2016). Written informed consent to participate in this study was provided by the participants' legal guardian/next of kin.

## Author Contributions

ST, TK, and KR conceptualized and planned the experiments. ST, TK, CK, MH, TG, MS, CE, JK, CT, and KR carried out the experiments. ST, TK, and CK contributed to sample preparation. ST, TK, CK, and TR contributed to the interpretation of the results. ST took the lead in writing the manuscript. All authors were involved in recruiting participants, provided critical feedback, and revised the manuscript.

## Conflict of Interest

The authors declare that the research was conducted in the absence of any commercial or financial relationships that could be construed as a potential conflict of interest.
